# Multi-omic characterization of the thermal stress phenome in the stony coral *Montipora capitata*

**DOI:** 10.7717/peerj.12335

**Published:** 2021-11-10

**Authors:** Amanda Williams, Jananan S. Pathmanathan, Timothy G. Stephens, Xiaoyang Su, Eric N. Chiles, Dennis Conetta, Hollie M. Putnam, Debashish Bhattacharya

**Affiliations:** 1 Microbial Biology Graduate Program, Rutgers University, New Brunswick, United States; 2 Department of Biochemistry and Microbiology, Rutgers University, New Brunswick, United States; 3 Department of Medicine, Division of Endocrinology, Robert Wood Johnson Medical School, Rutgers University, New Brunswick, United States; 4 Metabolomics Shared Resource, Rutgers Cancer Institute of New Jersey, Rutgers University,New Brunswick, United States; 5 Department of Biological Sciences, University of Rhode Island, Kingston, United States

**Keywords:** *Montipora capitata*, Metabolomics, Stress biology, Stony coral, Hawaii, Multi-omics analysis, Network analysis, Thermal stress, Transcriptomics

## Abstract

**Background:**

Corals, which form the foundation of biodiverse reef ecosystems, are under threat from warming oceans. Reefs provide essential ecological services, including food, income from tourism, nutrient cycling, waste removal, and the absorption of wave energy to mitigate erosion. Here, we studied the coral thermal stress response using network methods to analyze transcriptomic and polar metabolomic data generated from the Hawaiian rice coral *Montipora capitata*. Coral nubbins were exposed to ambient or thermal stress conditions over a 5-week period, coinciding with a mass spawning event of this species. The major goal of our study was to expand the inventory of thermal stress-related genes and metabolites present in *M. capitata* and to study gene-metabolite interactions. These interactions provide the foundation for functional or genetic analysis of key coral genes as well as provide potentially diagnostic markers of pre-bleaching stress. A secondary goal of our study was to analyze the accumulation of sex hormones prior to and during mass spawning to understand how thermal stress may impact reproductive success in *M. capitata*.

**Methods:**

*M. capitata* was exposed to thermal stress during its spawning cycle over the course of 5 weeks, during which time transcriptomic and polar metabolomic data were collected. We analyzed these data streams individually, and then integrated both data sets using MAGI (Metabolite Annotation and Gene Integration) to investigate molecular transitions and biochemical reactions.

**Results:**

Our results reveal the complexity of the thermal stress phenome in *M. capitata*, which includes many genes involved in redox regulation, biomineralization, and reproduction. The size and number of modules in the gene co-expression networks expanded from the initial stress response to the onset of bleaching. The later stages involved the suppression of metabolite transport by the coral host, including a variety of sodium-coupled transporters and a putative ammonium transporter, possibly as a response to reduction in algal productivity. The gene-metabolite integration data suggest that thermal treatment results in the activation of animal redox stress pathways involved in quenching molecular oxygen to prevent an overabundance of reactive oxygen species. Lastly, evidence that thermal stress affects reproductive activity was provided by the downregulation of *CYP-like* genes and the irregular production of sex hormones during the mass spawning cycle. Overall, redox regulation and metabolite transport are key components of the coral animal thermal stress phenome. Mass spawning was highly attenuated under thermal stress, suggesting that global climate change may negatively impact reproductive behavior in this species.

## Introduction

**Figure 1:**
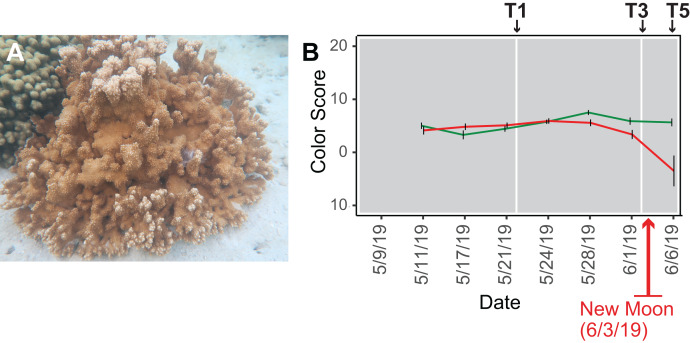
Analysis of the rice coral *Montipora capitata*. (A) *M. capitata* photographed in waters near the Hawaiʻi Institute of Marine Biology (HIMB) in O‘ahu, HI. Photo credit: Debashish Bhattacharya. (B) Color scores and their standard errors for the ambient (green line) and high temperature (red line) treated *M. capitata* nubbins that were cultured in tanks at HIMB. Low color scores indicate the bleaching phenotype in coral holobionts. The omics data sampling points are shown with the white lines at T1 (5/22/19), T3 (6/03/19), and T5 (6/07/19) (for details, see Williams et al. (2021)). The date of the New Moon in June 2019 is also shown.

Coral reefs are vitally important natural resources because they are home to about one-quarter of all marine biodiversity (Reaka-Kudla, 1997) and support an estimated one-half to one billion people living in coastal communities by providing food, income from tourism, and coastal protection (Woodhead et al., 2019). Since their radiation in the Middle Triassic period ~240 million years ago (Ma) (Veron, 1995), stony corals have survived five mass extinction events (Jackson, 2008). Their long-term survival underscores the inherent resilience of these holobionts in particular when considering the nutrient-poor marine environments in which they have thrived (Frankowiak et al., 2016). The coral holobiont (meta-organism) is comprised of the cnidarian animal host, algal symbionts, fungi, microbial aggregates, and viruses. Under ambient conditions, the algal cells can provide up to 100% of host energy needs in the form of lipids, carbohydrates, and amino acids, as well as excess O_2_ (Falkowski et al., 1984). In return, excess nitrogen and inorganic waste from the coral animal, namely water, ammonium, and CO_2_, are recycled by the algae, fueling cell metabolism (Yonge & Nicholls, 1931). Environmental shifts can lead to destabilization of the symbiosis (dysbiosis) between the coral animal and its partners because symbionts experience photo-oxidative stress and reduce provision of photosynthetic products. The coral animals may then expel their symbionts in the phenomenon known as “coral bleaching” (Muscatine & Porter, 1977). The target of our study, the hermaphroditic, broadcast spawning Hawaiian coral *Montipora capitata* (Fig. 1A), is a robust species that resists bleaching, even under conditions causing mortality in more susceptible species (Jokiel & Brown, 2004). The basis of bleaching resistance in *M. capitata* is yet to be fully explained but is most likely due to heterotrophic feeding (Grottoli, Rodrigues & Palardy, 2006).

We subjected *M. capitata* nubbins (coral fragments) to thermal stress over a 5-week period, during which time transcriptomic and polar metabolomic data were collected at three different time points (Fig. 1B). The period of sampling (late May to early June 2019) coincided with the first of three annual mass-spawning events for *M. capitata* in the region. Therefore, genes and metabolites involved in coral reproduction were expected to be present in the RNA-seq and polar metabolomics data. We studied genes of both known and unknown function (*i.e*., ‘dark’) and investigated the temporal dynamics and biological shifts that sustain the coral animal under heat stress. Dark genes are either novel or too highly diverged (BLASTP *e*-value cut-off ≤ 1e^−5^ against the nonredundant NCBI database) to identify putative homologs in existing data, although some may encode a known domain associated with novel sequence (Cleves et al., 2020). For example, 33% of dinoflagellate algal genes lack an annotation, but 1.4% of these unknown proteins contain a known domain (Stephens et al., 2018).

In our study, differentially expressed genes (DEGs) were filtered to only include reads which mapped to predicted *M. capitata* protein-coding genes (Shumaker et al., 2019): *i.e*., excluding algal RNA-seq reads. The animal data were integrated using networks to investigate molecular transitions in the coral. Network analysis can be a powerful framework for studying the structure of complex biological systems (Williams et al., 2021) with nodes representing units at all levels of the biological hierarchy and edges, interactions between them, including transcriptional control, biochemical interaction, energy flow, and species interactions. Usage of DEGs allowed us to focus on the most consequential gene expression changes. Modules containing known genes with known functions were used to investigate their roles in the thermal stress response, as well as to identify dark genes which provide interesting potential candidates for future gene knockout or knockdown experiments.

## Materials & Methods

### Culture conditions and sample collection

Our experimental design was previously described in Williams et al. (2021). Briefly, water was drawn from Kāne‘ohe Bay, O‘ahu and heated to 2.7–3.2 °C above ambient temperature (27–28 °C) in tanks at the Hawaiʻi Institute of Marine Biology (for details, see Williams et al., 2021) (Fig. S1). Given that *M. capitata* is a stress resilient coral, these conditions were designed to elicit a stress response in the coral, but not activate apoptotic (cell death) pathways. Coral nubbins from four colonies were fragmented so that each timepoint for both conditions had *n* = 3 nubbins. Nubbins were acclimated for 4 days after collection from Kāne‘ohe Bay before temperature ramp-up was initiated. The temperature in the heat stressed tanks was increased 0.4 °C every 2 days until they were between 2.7–3.2 °C above the ambient water temperature. Samples were collected at five time points (T1-5; Fig. 1B) during the 5-week experiment (Fig. S1). Samples from T1, T3, and T5 were chosen for RNA-seq analysis because they represent stress treatments after temperature ramp-up was complete, the point where bleaching begins (13 days after T1), and the last day of the 5-week period (17 days after T1), respectively (see Fig. 1B). The samples were flash frozen in liquid nitrogen upon collection and divided for RNA-seq and polar metabolomic analysis. Four colonies were used for metabolomic analysis but only one (genotype 289) was used to prepare cDNA libraries. This approach led to 11–13 individual samples per time point in the metabolomic analysis with three nubbins (sometimes two or four) representing each genotype (see Williams et al., 2021). Approval to collect coral nubbins from the waters of Kāne‘ohe Bay, HI was provided by the Division of Aquatic Resources, State of Hawaiʻi under SAP 2019-60.

### Color scores

Color scores, an accepted proxy for bleaching progression, were recorded for the ambient and stress treated nubbins at each of the five time points (Fig. 1B; Siebeck et al., 2006). Nubbins were photographed next to a red/blue/green color standard. ImageJ was used to extract red/blue/green values from the color standard and each nubbin in the tanks. Dividing the experimental value observed in the nubbins by the corresponding color standards allowed the coral values to be standardized (Edmunds, Gates & Gleason, 2003). Bleaching scores were quantified as PC1 from principal component analysis of these data using the normalized intensity values from each color channel (Williams et al., 2021).

### Polar metabolite processing

Polar metabolite extractions were based on Lu et al. (2017). In a glass Dounce homogenizer, samples were mechanically ground in one mL of 40:40:20 (methanol:acetonitrile:water) (v/v/v) + 0.1 M formic acid extraction buffer after incubation in the buffer for 5 min. The sample was transferred to a 1.5-mL Eppendorf tube, with an additional 500 μL of extraction buffer used to rinse the Dounce. The samples were then vortexed for 10 s, before a 10-min centrifugation (16,000*g*) at 4 °C. A total of 500 μL of the homogenate was then transferred to another Eppendorf tube and 44 μL of 15% NH_4_HCO_3_ was added to neutralize the extraction buffer.

The samples were run on an ultra-high–performance LC-MS (UHPLC-MS), consisting of a Vanquish Horizon UHPLC system (Thermo Fisher Scientific, Waltham, MA, USA) with XBridge BEH Amide column (150 mm by 2.1 mm, 2.5-μm particle size; Waters, Milford, MA, USA), and a Thermo Fisher Scientific Q Exactive Plus with a HESI source. The solvent and run conditions for both the UHPLC and the MS are described in Williams et al. (2021), along with an in-depth metabolite extraction protocol.

### Metabolite data

Metabolomic data for the time points analyzed in this study were published by Williams et al. (2021) and are available as supplementary information associated with the manuscript (http://advances.sciencemag.org/cgi/content/full/7/1/eabd4210/DC1).

### cDNA library preparation

Total RNA was extracted using liquid nitrogen and a mortar and pestle. RNA was isolated with the Qiagen AllPrep DNA/RNA/miRNA Universal Kit and strand specific cDNA libraries prepared using the TruSeq RNA Sample Preparation Kit v2 (Illumina) following the manufacturer’s instructions. This protocol includes poly-A selection to target eukaryotic cells, eliminating reads from the prokaryotic microbiome. Quality control for the libraries was done using an Agilent Bioanalyer, with library length being ~250 bp. Sequencing was performed on the NovaSeq (2 × 150 bp) by the vendor GeneWiz. These RNA-seq data are available under NCBI BioProject ID: PRJNA694677 (see also Table S1).

### RNA-seq preprocessing

RNA-seq reads were trimmed using Trimmomatic v0.38 (mode ‘PE’; ILLUMINACLIP:adapters.fasta:2:30:10 SLIDINGWINDOW:4:5 LEADING:5 TRAILING:5 MINLEN:25) (Bolger, Lohse & Usadel, 2014), only pairs for which both mates remained after trimming were used for subsequent analysis.

### Functional annotation

The reference *M. capitata* proteins were annotated using the Uniprot database (release 2020_06). BLASTP (version 2.7.1+, parameters: *e*-value 1e^−5^-seg yes -soft_masking true and pident ≥ 30%) was used to query the predicted proteins against the Uniprot (SwissProt + TrEMBL) protein database. Function assignment was based on the best hit criterion. Proteins without hits against Uniprot or annotated as “Unknown” were compared using BLASTP against the current NCBI nr database.

### Differentially expressed genes (DEGs)

Expression of the available *M. capitata* genes (Shumaker et al., 2019) over the sequenced timepoints was quantified using Salmon v1.10 (–allowDovetail –validateMappings –seqBias –gcBias) (Patro et al., 2017). We retained genes with a TPM value ≥ 5 in each sample. The R-package DESeq2 (Love, Huber & Anders, 2014) was used to find the DEGs by comparing the ambient *versus* stressed condition for each time point. Genes of interest, identified as being differentially expressed between the ambient *versus* high temperature treatments, were further analyzed by checking for differential expression between T1 and T3, and T3 and T5 for both thermally stressed and ambient samples. An adjusted *p*-value of ≤ 0.05 and log2-fold change (FC) ≥ 1 was used for initial filtering of differential expression results.

### Co-expression networks

The R-package DGCA (McKenzie et al., 2016) was used to determine the correlation between pairs of genes respectively for each time point. The pairwise correlation was calculated with the function matCorr using Pearson method. The functions matCorSig and adjustPVals were used to calculate and adjust (with the Benjamini–Hochberg method) the correlation *p*-values, respectively. Only pairs with an adjusted *p*-value ≤ 0.05 were used to construct the co-expression networks. Module detection was done using the functions hclust (method = “average”) and cutreeDynamicTree (minModuleSize = 10 and deepSplit = TRUE). Modules were labeled manually based on our interpretation of the data.

### Differentially accumulated metabolites (DAMs)

The R-package mixOmics (Rohart et al., 2017) was used to detect differentially accumulated metabolites **(**DAMs) (VIP score ≥ 1 and FC ≥ 2). The code used for the DAM + gene/metabolite co-expression networks have been submitted to https://github.com/dbsymbiosis/Construct_Networks.

### Data integration

To integrate the gene-metabolite data, we used MAGI (Metabolite Annotation and Gene Integration; Erbilgin et al., 2019) because it is suited for non-model organisms. In these taxa, gene annotations are based on bioinformatic transfer of function and gene membership in many well-characterized biochemical pathways are unvalidated. Coupling metabolomic and genome-wide gene expression data in challenging models such as corals provides a basis for improving the annotation of both types of data and a way to meaningfully interpret observed trends. Briefly, MAGI uses a biochemical reaction network to numerically score the provided Liquid chromatography-Mass Spectrometry (LCMS) features (Liu & Locasale, 2017) and protein or gene sequences provided by the user. The putative compound identification and input sequences are connected to biochemical reactions by a chemical similarity network and evaluated based on sequence homology against a reference database (Erbilgin et al., 2019). The likelihood of identifying an LCMS feature/gene function increases if there is a gene function/metabolite feature to substantiate that metabolite identity/gene function. Therefore, MAGI leverages the association between genes and metabolites to create higher quality annotations for both datasets. The MAGI score is a geometric mean of the homology score, reciprocal score, reaction connection score, and compound score, representing the probability and strength of the gene-metabolite association.

The metabolic features given to MAGI were defined using the mass-to-charge ratio (m/z) and retention time (rt). The MAGI results were filtered, whereby only DAM-DEG connections with a compound_score = 1, e_score_r2g (reaction-to-gene) > 5, e_score_g2r (gene-to-reaction) > 5, and reciprocal_score = 2 were retained. A stringent e_score_g2r and e_score_r2g cut-off of ≥ 5 was used to ensure reliability of the connections between DAMs and DEGs. We checked each reaction manually for DAMs and DEGs of interest.

## Results

### The early stress response

Because there was an unexpected warming event in Kāne‘ohe Bay during the experiment that increased the ambient seawater temperature by ca. 2 °C (Williams et al., 2021), we expected the gene co-expression data to show evidence of thermal stress at T1, that should become more pronounced at T3 and T5. The color scores for *M. capitata* nubbins do not reflect this prediction of stress at T1, likely due to the high stress resistance of *M. capitata* (Fig. 1B), however, lower color scores and partial bleaching are apparent at T5. The network statistics reflect the temporal growth in complexity of the stress response (Table S2).

**Figure 2:**
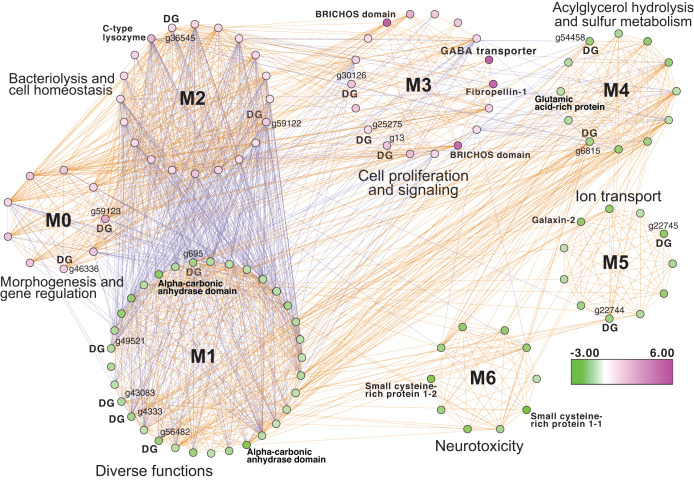
Gene co-expression analysis of *M. capitata*. Network of differentially expressed genes in *M. capitata* at TP1 (the early thermal stress phenome) showing the different gene modules and their interactions. Purple nodes are up-regulated and green nodes are down-regulated. All dark genes are marked with DG with *M. capitata* gene IDs shown. The down-regulated genes in M6 that are dominated by members of the small cysteine-rich protein family, often involved in signaling and protein interactions, are annotated. Only selected genes are annotated in this network and module annotations provide a representation of overall function(s). The annotations of all genes (when known) in each module in this, and all networks generated by this study, are available in File S1 (Cytoscape file).

Inspection of the full network of DEGs at T1 shows differential regulation of a limited number of stress pathways (Fig. 2; File S1 (Cytoscape file)). The most strongly upregulated candidates are in module M3 and include genes involved in molecular chaperone functions such as brichos domain-containing proteins (*M. capitata* gene g29710, fold-change (FC) = 5.94; g29707 FC = 5.40) and a previously described protein in sea urchin and sea cucumber that is involved in embryo development (fibropellin-1, g71193, FC = 5.47; Bisgrove, Andrews & Raff, 1995; Ba et al., 2015). Interestingly, fibropellin-1 gene family expression remains upregulated but at a much lower fold-change at T5 (see below) that follows the mass spawning event of *M. capitata* (g30756 FC = 1.49; g30753 FC =1.37). Within M2 in the T1 network are well-characterized genes such as C-type lysozyme that is involved in bacteriolysis and the immune response (g29445 FC = 2.41; Ragland & Criss, 2017). This gene has the highest degree value (56) in the T1 network (*i.e*., number of edges linked to a node) which indicates that it acts as an important regulatory component of the transcriptional network (Shumaker et al., 2019). Genes with a potential role in biomineralization, a glutamic acid-rich protein (adi2mcaRNA37907_R0, FC = −1.12) and galaxin-2 (g25962, FC = −2.04), whose products are associated with the coral skeletal organic matrix (SOM) (Conci, Wörheide & Vargas, 2019), are weakly to moderately downregulated in modules M4 and M5, respectively. Also occupying key positions in the T1 network are dark genes that are marked as “DG” in Fig. 2, with gene numbers shown. We highlight *M. capitata* dark gene g36545 that has a weak hit to a N-terminal death-domain superfamily (*e*-value = 6.79e^−04^) and a high degree value = 43. Analysis of distribution demonstrates that dark gene g36545 is shared by, and limited, to other stony corals (Fig. S2).

### Downregulated genes in the later stress response

**Figure 3:**
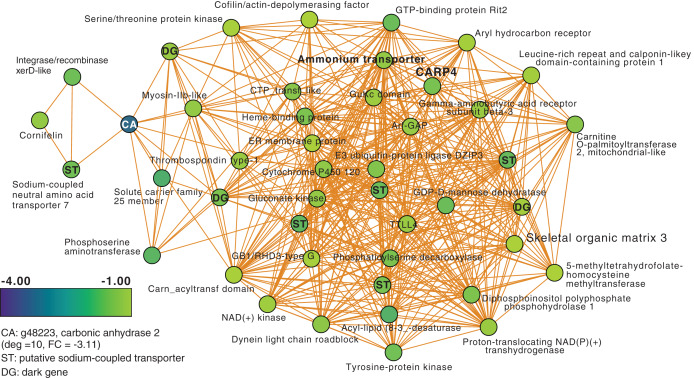
*M. capitata* TP5 module M1 of significantly down-regulated genes that includes many transporters. The legend for level of downregulation is shown. Dark genes are identified with DG and genes encoding sodium-coupled transporters are marked with ST.

Next, we focused on the networks generated from the T3 and T5 DEG data for *M. capitata*. These networks are larger than the T1 network; each comprising 20 modules (Fig. S3). We identified some genes with high degree in these networks, as well as dark genes, but will focus here (not exhaustively) on individual modules with previously well-characterized thermal stress response genes in the T5 network to gain insights into the later stage of the thermal stress response. M1 in the TP5 network (Fig. 3) contains many significantly downregulated genes that are dominated by metabolite transporters. These include a variety of sodium-coupled transporters (ST), including a sodium-coupled neutral amino acid transporter (gene adi2mcaRNA35257_R0, FC = −1.61), a sodium-coupled monocarboxylate transporter 1 (g37389 FC = −1.53) putatively involved in the transport of a variety of substrates including short-chain fatty acids and lactate (Song et al., 2020), and a probable sodium/potassium-transporting ATPase subunit (g39446 FC = −1.68) involved in the sodium-coupled active transport of nutrients (Song et al., 2020). The transporter with the highest degree (deg) in this module (deg = 36) is a putative ammonium transporter that is weakly downregulated (g26425, deg = 36, FC = −1.29; Fig. 3).

Another key component of module M1 is the skeletal aspartic acid-rich protein 3 that is related to coral acid-rich protein 4 (CARP4; ca. 40% protein identity) involved in biomineralization (CaCO_3_, aragonite in corals). CARPs are largely independently derived, secreted proteins rich in glutamic and aspartic acid residues that accumulate in the calicoblastic space of corals, playing roles in calcification (Drake et al., 2013; Guzman et al., 2018; Peled et al., 2020; Levy et al., 2021). CARP-encoding genes are differentially expressed during coral development with CARP4 and CARP5 strongly up-regulated in the calcifying spat stage of *Pocillopora damicornis* (Mass et al., 2016; Bhattacharya et al., 2016). In M1, a single CARP is present, that is centrally located in the network (deg = 21) and downregulated at TP5 (FC = −1.53). A maximum likelihood phylogeny of this protein (Fig. S4) shows this gene to be present in non-coral species and to have undergone ancient gene duplications (provisionally named C1–C4 and R1–R4 for complex and robust coral species, respectively) within the scleractinian lineage with *M. capitata* encoding divergent paralogs. However, only the gene (g43402) encoding CARP4 is significantly downregulated under thermal stress in this species. These results indicate that the *M. capitata* thermal stress phenome includes suppression of the biomineralization reaction (also evident in TP1, see above) with the concomitant down-regulation of a putative carbonic anhydrase 2 (Fig. 3) that is the most highly downregulated gene in M1 (g48223 FC = −3.11). This zinc metalloenzyme catalyzes the reversible hydration of carbon dioxide to bicarbonate (Bhattacharya et al., 2016).

### Up-regulated genes after 5 weeks of thermal stress

**Figure 4:**
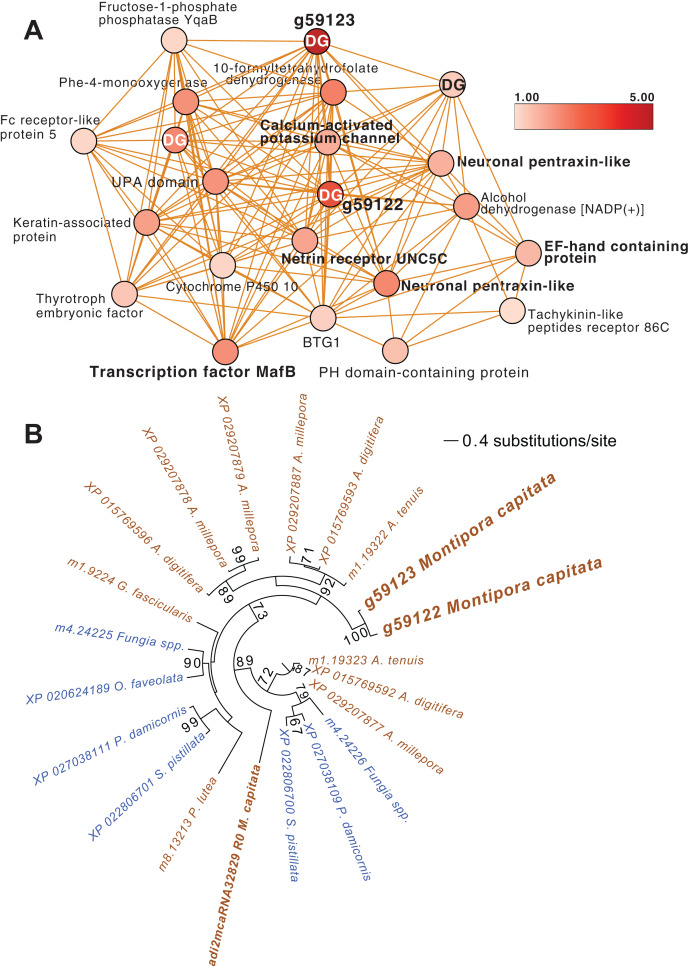
*M. capitata* TP5 module M4 of significantly upregulated genes. (A) The legend for level of upregulation is shown. Dark genes are identified with DG. (B) Maximum likelihood (IQ-Tree; Trifinopoulos et al., 2016) phylogenetic analysis of paralogous coral dark genes g59122 and g59133 and related homologs inferred using default parameters and 1,000 ultrafast bootstrap replicates. The results of the bootstrap analysis are shown on the branches when >60%. The legend shows the expected substitution rate for the protein dataset. Complex and robust coral species are shown in brown and blue text, respectively.

Another module of interest in TP5 is M4 (Fig. 4A), that contains a variety of significantly up-regulated genes with roles in signaling and immunity (*e.g*., netrin receptor UNC5C (g6679 FC = 2.00) and two neuronal pentraxin-like genes (g46559, g46566 FC = 1.79, 2.45, respectively)) and transcriptional regulation (*e.g*., BTG1 protein (g32300 FC = 1.27), MafB (g30496 FC = 2.36) and thyrotroph embryonic factor (g57753 FC = 1.42)). BTG family members are transcriptional regulators that can enhance or repress the activity of transcription factors. Maf proteins are widespread among metazoans, including corals, and are bZIP (basic (DNA-binding) and leucine zipper (homo- or heterodimerization) domains) transcriptional factors that are involved in oxidative stress and detoxification pathways (Kannan, Solovieva & Blank, 2012; Shinzato et al., 2012). Multiple Maf genes are upregulated at TP5 in M2, including *mafF* (g30493 FC = 2.39) and two Maf domain-containing proteins (g30494, g30495 FC = 1.96, 2.04, respectively). Two Maf domain-containing proteins are present in M18 (g2209, g26625 FC = 1.41, 1.24, respectively). In M4, the pentraxin domain-containing proteins are of interest because these are multimeric, calcium-binding proteins often involved in immunological responses (Ma & Garred, 2018). Located in this module are two proteins that interact with calcium: one is a calcium-binding EF-hand protein (g14108 FC = 1.67) and the second is a calcium-activated potassium channel subunit (g16479 FC = 1.86).

Embedded within this network of conserved stress response proteins are four dark genes, two of which are paralogs that comprise highly connected hubs in this module (g59122 and g59123, both have deg = 18 and lack a domain hit using CDSEARCH). This gene family was only detected in stony corals (Fig. 4B) and offers a promising target for functional analysis. These dark genes show a high fold-change in gene expression when compared to ambient conditions (g59122, FC = 3.55) with g59123 having the highest value in this module (FC = 4.48).

## Gene-metabolite interactions

### Animal response to redox stress

Analysis of the MAGI output provided clear evidence of redox stress in the coral animal (Table 1), with 21/27 of the high-confidence upregulated reactions at T5 having oxidoreductase functions (Table S3). Of these 21 reactions, 10 involve O_2_ as a substrate and the release of a water molecule, the majority of which include cytochrome P450 domains. The rate of metabolism at higher temperatures increases and can lead to physiological hyperoxia. Under elevated temperatures, oxygen absorbs excitation energy and becomes active in the form of superoxide radicals and hydrogen peroxide (Lesser, 1997). These reactive oxygen species (ROS), which are likely to be key contributors to coral thermal stress (Cziesielski et al., 2018; Cleves et al., 2020), derive their reactivity from the unpaired electron. Hence, the enrichment of oxidoreductases is an expected outcome. Their catalysis solely involves the transfer of electrons; therefore, we postulate that corals utilize oxidoreductases to maintain redox homeostasis, remove excess molecular oxygen, and thereby, limit the production of ROS.

**Table 1:** Results of the MAGI analysis.

Gene annotation	Compound name	MAGI score	Reaction
Putative alanine-glyoxylate aminotransferase agt2	Acetaldehyde	10.63	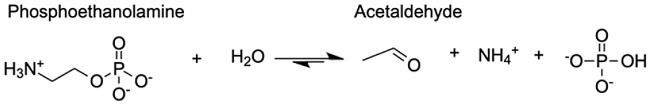
Aldo-keto reductase family 1 member A1	(E)-2-hexenal	8.73	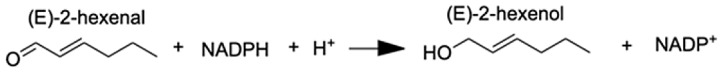
Aldo-keto reductase family 1 member A1	Acrolein	8.68	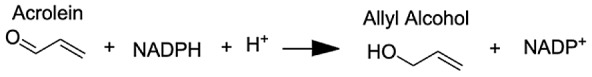
Aldo-keto reductase family 1 member A1	5α-androstane-3 β,17 β-diol	9.05	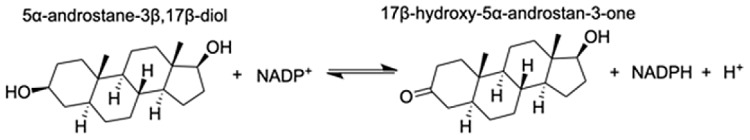
Aldo-keto reductase family 1 member A1	L-gulonic acid	7.71	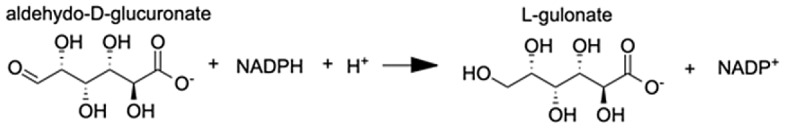
Phenylalanine-4-hydroxylase	L-phenylalanine 4α-hydroxy-tetrahydropterin L-tyrosine	10.80 10.91 11.49	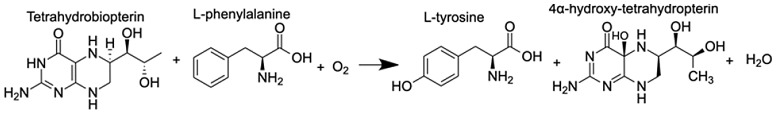
Cytochrome P450 1A1-like	Progesterone	6.31	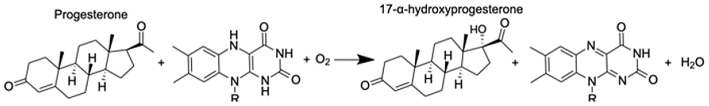
Cytochrome P450, family 3, subfamily A, polypeptide 4	1,8-cineole	8.44	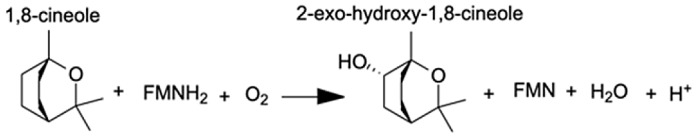
Cytochrome P450 27C1-like isoform X3	(25R)-3α,7α,12α-Trihydroxy-5β-cholestan-26-al	6.6	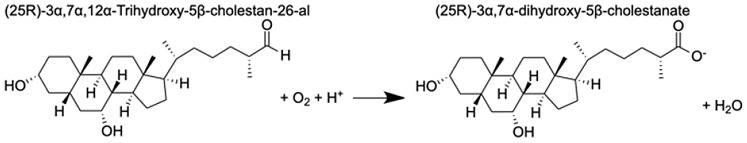
Flavin-containing monooxygenase	N,N-dimethylaniline N-oxide	11.2	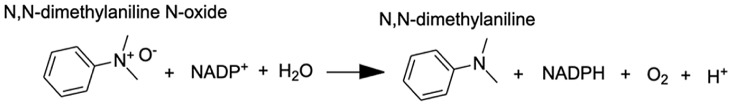
Inositol oxygenase	D-glucuronic Acid	7.26	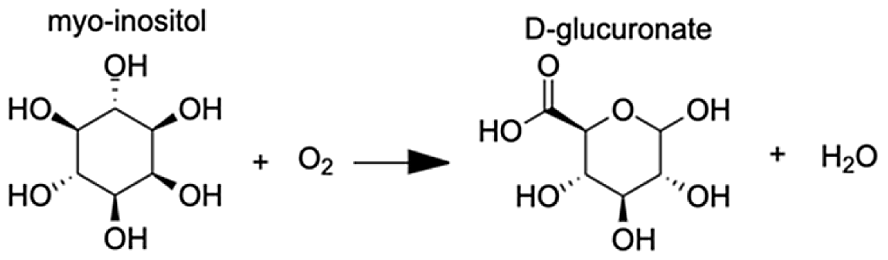
Delta-1-pyrroline-5-carboxylate dehydrogenase, mitochondrial	4-hydroxyglutamate semialdehyde	11.23	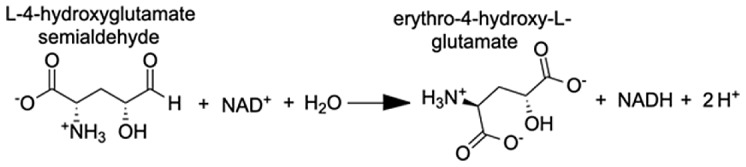
Glyoxylate reductase hydroxypyruvate reductase	(2R)-2,3-dihydroxypropanoic acid (Glycerate)	9.97	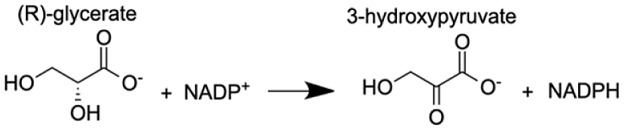

**Note:**

Pathways with the highest MAGI scores that are upregulated under thermal stress at TP5 are shown.

### Upregulation of the phenylalanine-4-hydroxylase pathway

A pathway of particular interest with regard to the coral thermal stress response involves phenylalanine-4-hydroxylase (P4H), which is a homotetramer of four phenylalanine hydroxylase (PH) enzymes, each containing three domains (a regulatory N-terminal domain, a catalytic domain, and a C-terminal domain) that use a non-heme Fe(II) cofactor (Fitzpatrick, 1999). P4H catalyzes the bidirectional reaction of L-phenylalanine to L-tyrosine with (6*R*)-L-*erythro*-5,6,7,8-tetrahydrobiopterin (BH_4_) as a cofactor (Table 1). The gene expression and metabolite integration results show upregulation of the *p4h* gene (FC = 1.27), as well as increased ion counts for all reaction participants except BH_4_. BH_4_ donates two electrons to reduce the iron atom to ferrous iron and cleaves O_2_ to reduce phenylalanine (Phe) to tyrosine (Tyr). Molecular oxygen can oxidize the ferrous iron, regenerating the enzyme. In this pathway, 4α-hydroxy-tetrahydropterin is first dehydrated and then reduced by an NADH-dependent component of P4H, the phenylalanine hydroxylase stimulator (PHS) (Lei & Kaufman, 1998). Phe and Tyr are both synthesized by scleractinian corals, either from intermediates in glycolysis, gluconeogenesis, the pentose phosphate pathway (PPP), the tricarboxylic acid cycle (TCA), or the pentose phosphate shunt, depending on the substrate used in previous studies (Fitzgerald & Szmant, 1997). When coral samples are incubated with ^14^C lysine, Tyr and Phe are produced through gluconeogenesis, glycolysis, or the PPP following the TCA cycle. Corals can also take up dissolved free amino acids from surrounding sea water (Ferrier, 1991). These results could explain the lack of reactant depletion in the P4H pathway. Although P4H can function bidirectionally, it is more likely that the enzyme is reducing Phe to Tyr. The reverse reaction is not energetically favored because P4H preferentially binds Phe rather than Tyr, and one of the most important biological roles of Phe is producing Tyr, a substrate for receptor tyrosine kinases that are implicated in the coral stress response (Bellantuono et al., 2012).

### Dysregulation of spawning during thermal stress

**Figure 5:**
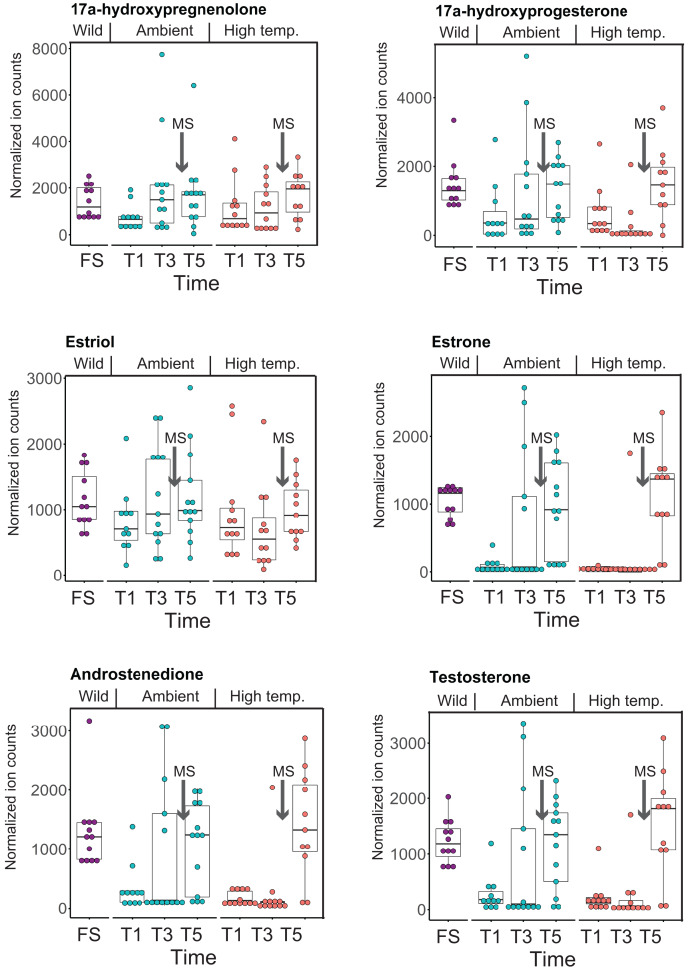
Analysis of sex steroids in *M. capitata*. Accumulation of a variety of predicted sex steroids in *M. capitata* nubbins over the duration of the ambient and thermal stress treatments as well as from wild populations collected after T5 from the same colonies used in the tank experiments (FS; for details, see Williams et al., 2021). Each dot represents a single nubbin measurement from four different genotypes (2–4 nubbins (usually 3)/genotype were sampled). The pattern of metabolite accumulation suggests that these steroid levels increased at T5 (Fig. 1B), which preceded the expected mass spawning event (arrow labeled with “New Moon” between T3 and T5) for this species in June 2019. The putative functions of these steroids are as follows: 17a-hydroxypregnenolone–a neuromodulator generated by the action of mitochondrial cytochrome P450 enzyme 17α-hydroxylase (CYP17A1) that is an intermediate in the delta-5 pathway of biosynthesis of gonadal steroid hormones and adrenal corticosteroids; 17a-hydroxyprogesterone–progestogen that is a chemical intermediate in the biosynthesis of androgens, estrogens, glucocorticoids, and mineralocorticoids; estriol–female sex hormone (weak estrogen), with a large amount produced in humans by the placenta; estrone–another female sex hormone (weak estrogen), binds to the estrogen response element and promotes transcription of genes involved in the female reproductive system functions; androstenedione-weak androgen steroid hormone, precursor of testosterone and other androgens; testosterone-primary male sex hormone involved in development of male reproductive tissues.

*CYP-like* genes, which facilitate the biotransformation of important intracellular compounds (Lu et al., 2020), are implicated in several pathways at TP5 in our MAGI results (Table 1). One of these involved the downregulation of progesterone and a *CYP-like* gene (FC = −1.10) during the *M. capitata* spawning period. Beyond the MAGI results regarding progesterone, analysis of existing metabolite ion counts from untargeted UHPLC-MS analysis of *M. capitata* (Williams et al., 2021) shows that predicted sex steroids in this species follow the expected increase in accumulation (*e.g*., testosterone, estrone, androstenedione) under ambient conditions during the mass spawning event (Fig. 5).

## Discussion

Coral reefs are under worldwide threat from warming oceans and local human-caused stressors such as over-fishing, the discharge of pollutants, and uncontrolled development (National Academies of Sciences, Engineering, & Medicine, 2019). In response, many advances have been made in identifying individual gene and metabolite markers of coral thermal stress (National Academies of Sciences, Engineering, & Medicine, 2019), but little has been done to link these two omics data sources. This is explained by the complexity of holobiont metabolomic interactions, combined with the massive number of dark genes and dark metabolites in corals for which currently no function, and therefore no causal relationship exists (Williams et al., 2021). In addition, because metabolites are shared among holobiont members, obscuring metabolite origin, it is challenging to make biologically meaningful predictions from these data alone. For this reason, we used MAGI to find links between polar metabolite accumulation and gene expression. This approach provides a foundation for studying non-model systems by exploiting the consensus between metabolite identification and gene annotation to generate metabolite-gene associations (Erbilgin et al., 2019). The MAGI analysis revealed the heightened response of the coral animal to redox stress, including the scavenging of excess molecular oxygen. The rate of metabolism at higher temperatures increases and can lead to physiological hyperoxia. Under elevated temperatures, oxygen absorbs excitation energy and becomes active in the form of superoxide radicals and hydrogen peroxide (Lesser, 1997). These ROS, which are likely to be key contributors to coral thermal stress (Cziesielski et al., 2018; Cleves et al., 2020), derive their reactivity from the unpaired electron. Hence, the enrichment of oxidoreductases is an expected outcome. Their catalysis solely involves the transfer of electrons; therefore, we postulate that corals utilize oxidoreductases to maintain redox homeostasis, remove excess molecular oxygen, and thereby, limit the production of ROS.

In addition, we find evidence that progesterone metabolism may be implicated in the unsynchronized mass spawning events that have occurred at the study site in recent years (Fogarty & Marhaver, 2019). Progesterone, a sex steroid, can be produced multiple ways, but usually involves *β*-hydroxylation reactions catalyzed by CYP enzymes (Lu et al., 2020). Many examples of CYP enzymes metabolizing progesterones occur in metazoans (Baker, 2001), such as CYP1A in humans (Lu et al., 2020) and CPY 17 dehydrogenase (CYP17) in scleractinian corals (Rougée, Richmond & Collier, 2015; Blomquist et al., 2006). There is evidence that sex steroids may regulate scleractinian reproduction (Tarrant, Atkinson & Atkinson, 1999). CYP17 converts progesterone to androgens and Rougée, Richmond & Collier (2015) found that in the absence of thermal stress the enzymatic activity of CYP17 remained consistent over the lunar cycle in the brooding coral *Pocillopora damicornis* (Twan et al., 2006). Twan, Hwang & Chang, 2003 found that the production of androgens increased prior to spawning in *Euphylia ancora*. The dysregulation of coral spawning due to environmental stress has been reported (Fogarty & Marhaver, 2019) and occurred during the first mass spawning event for *M. capitata* around O‘ahu, HI in June 2019. Therefore, our results indicate that thermal stress, among other functions, affects the production of hormones contributing to reproductive activity.

One of the most notable findings of the co-expression network analysis is that they are dominated by downregulated metabolite transport genes. The suppression of metabolite transport by the coral host may potentially be a response to reduction in algal productivity. More likely, it indicates redox stress, resulting from the animal host and/or algal symbionts, which leads to the generation of reactive species due to dysfunction in electron transport (see below; Roberty, Furla & Plumier, 2016). The inhibition of organic carbon production by the algae, precipitated by prolonged thermal stress (Hillyer et al., 2017), can ultimately lead to their expulsion, resulting in bleaching (Slavov et al., 2016). That is, in addition to a role in host processes, the coral animal may be dampening algal proliferation by reducing access to nutrients needed for growth such as ammonium, as demonstrated in the cnidarian model *Aiptasia* under the symbiotic stage (Cui et al., 2019). This hypothesis conflicts with the findings of Fernandes de Barros Marangoni et al. (2020) who found that ammonium enrichment reduced thermal stress in the coral *Stylophora pistillata* and supported symbiont stability. This aspect may be less important for Hawaiian *M. capitata* that can meet 100% of its energy needs through heterotrophic feeding during periods of bleaching (Grottoli, Rodrigues & Palardy, 2006).

Our study provides important advances in the areas described above, however, three aspects of the results deserve further discussion. The first is the gene-metabolite interaction analysis of the phenylalanine-4-hydroxylase pathway in which BH_4_ was unexpectedly absent in the MAGI results. Some plausible explanations for this result are as follows. In the kinetic model, P4H requires BH_4_, Phe, and O_2_ to be bound, in that order (Volner, Zoidakis & Abu-Omar, 2003). BH_4_ binds first, converting the enzyme to its inactive form, E_i_, until sufficient Phe is present in plasma, at which point Phe binds and converts P4H to its activated form, E_a_ (Xia, Gray & Shiman, 1994); BH_4_ bound to P4H would not have been detected in our analysis. Given that BH_4_ is involved in other cellular functions it is possible that its levels might be depleted under heat stress, despite upregulation of the P4H pathway. This is relevant when considering the stoichiometry of the reaction, specifically, the number of BH_4_ molecules needed as cofactors depends on cellular conditions. Higher pH and temperature may require more than one BH_4_ to reduce two iron atoms (Fitzpatrick, 2003), further reducing the number of free BH_4_ molecules available for detection. It is also possible that another tetrahydropterin was used as an electron donor because BH_4_ is primarily used to combat oxidative stress (Kraft et al., 2019), potentially limiting its supply during high temperature stress. Existing data demonstrate the likely involvement of P4H in the symbiotic relationship between *Hydra viridissima* and its photosymbiont *Chlorella* sp. A99 (Hamada et al., 2018).

The second aspect is the impact of thermal stress on the coral reproductive cycle. Inspection of Fig. 5 shows that sex steroid accumulation is generally reduced under thermal stress, however, at T5, recover to near ambient and wild sample levels for several compounds (*e.g*., estrone, androstenedione, testosterone). This suggests that *M. capitata* may be able to partially acclimate to warming waters vis-à-vis sex steroid production, although these preliminary results need validation. More broadly, our results demonstrate that thermal stress impacts the production of hormones linked to reproductive activity. It is likely that the negative impact of environmental stress on coral mass spawning events will become more prevalent as oceans become warmer. Despite this not being the original intent of our study, the data we have generated provides valuable insights into how thermal stress disturbs the reproductive cycle of broadcast spawning corals. The consequences of this disturbance may have profound impacts not only on the health of existing reef ecosystems, but also on the ability of coral reefs to recover and recolonize an area after a major bleaching event or any environmental disturbance. The combined impact of thermal stress and mass spawning were addressed in our study, and it is possible that their interactions make it more difficult to interpret thermal stress impacts in isolation. Peak bleaching occurs in Hawaiian *M. capitata* in the month of October when the water temperatures are at their highest (Cunning, Ritson-Williams & Gates, 2016). However, as our study in 2019 demonstrated (Williams et al., 2021), local warming events can occur during mass spawning periods and will impact coral reproduction (current data). Therefore, rather than being weakened by the co-occurrence of warming and spawning in our study corals, we consider our data to be important for understanding how these combined stresses may impact future coral health as local warming events, like those we encountered, become more frequent.

Finally, it should be noted that although the interaction between the coral and its algal endosymbionts represents the cornerstone of reef ecosystems, we chose to target the host animal response to thermal stress in this study. Whereas the metabolomic data analyzed here is derived from the whole holobiont (*i.e*., coral, algae, and other microbiome components), the RNA-seq data only captured transcripts from the eukaryotic component (*i.e*., coral and algae). The integration of the algal data was hindered by the lack of reference genomes for the endosymbionts of *M. capitata* and the likely presence of cryptic eukaryotic components of the holobiont that might contribute to non-animal RNA-seq data (Kwong et al., 2019). A recent paper demonstrated that Symbiodiniaceae genomes are highly diverged, even between species in a single genus (*Symbiodinium*; González-Pech et al., 2021) and that multiple algal symbionts from different genera may reside in a single coral nubbin. Furthermore, metabolomes of the host and symbiont are not affected by variation in the abundance of the two algal symbionts that dominate Hawaiian *M. capitata* colonies (*i.e*., *Durusdinium glynnii* and *Cladocopium* spp.) (Matthews et al., 2020). For these reasons, we concluded that the host response to thermal stress, reflecting the holobiont contribution, was the best target for this poorly characterized coral model. The results presented here provide a foundation upon which questions regarding coral-algal interactions during stress can be addressed in future studies.

## Conclusions

This work contributes to our understanding of the coral response to thermal stress and the potential effects that a warming ocean will have on the reproductive health of these organisms. The early thermal stress response of *M. capitata* involves the downregulation of growth and DNA replication and the upregulation of signaling and the immune response. Later stages show downregulation of metabolite transport and biomineralization, as well as an upregulation of transcriptional regulators. Activation of animal redox stress pathways potentially as a mechanism for the detoxification of reactive oxygen species was found to be a major outcome of thermal stress. Whereas there was a noticeable increase in sex hormones (*e.g*., progesterone) in our samples prior to a natural mass spawning event, the release of egg-sperm bundles by *M. capitata* was highly attenuated in June 2019 (DB, HMP unpublished data), suggesting that thermal stress may negatively impact the reproductive behavior in this species. Significant effort will be needed to modify this polygenic trait in coral holobionts to boost resilience to thermal stress in the long term. Nonetheless, we have identified several novel genes that are promising candidates for functional analysis using the recently developed CRISPR/Cas9 tools for corals (Cleves et al., 2018; Cleves et al., 2020). It is important to remember that the algal symbionts of corals play a key role in holobiont biology and stress response vis-à-vis symbiotic nutrient cycling (Rädecker et al., 2021). Therefore, future gene-metabolite interaction analyses need to address *in situ* algal gene expression to address the integration of the host-symbiont response to thermal stress.

## Supplemental Information

10.7717/peerj.12335/supp-1 Supplemental Information 1 Temperature profiles for the ambient and high temperature treatments done at the Hawaiʻi Institute of Marine Biology (HIMB). Ambient temperature tank profiles are shown in variations of blue and high temperature tanks in variations of red. Vertical black lines indicate sampling points T1, T3, and T5 (see Methods).

10.7717/peerj.12335/supp-2 Supplemental Information 2 Maximum likelihood (IQ-Tree) phylogenetic analysis of *M. capitata* dark gene g36545 done using default parameters and 1000 ultrafast bootstrap replicates. The results of the bootstrap analysis are shown on the branches when >60%. The legends show the expected substitution rate for the protein dataset.

10.7717/peerj.12335/supp-3 Supplemental Information 3 *M. capitata* networks of differentially expressed genes at TP3 (top) and TP5 (bottom) showing the different gene modules and their interactions. Red nodes are up-regulated, green nodes are down-regulated, and selected dark genes are the yellow nodes with gene IDs shown. The fold change (FC) and network degree value (deg) are also shown for some genes.

10.7717/peerj.12335/supp-4 Supplemental Information 4 Maximum likelihood (IQ-Tree) phylogenetic analysis of coral CARP5 homologs inferred using default parameters and 1000 ultrafast bootstrap replicates. The results of the bootstrap analysis are shown on the branches when >60%. The legend shows the expected substitution rate for the protein dataset. Complex and robust coral species are shown in brown and blue text, respectively. Four putative CARP5 paralog clades are indicated. The thick branches mark a major gene duplication event in the common ancestor of complex and robust coral species.

10.7717/peerj.12335/supp-5 Supplemental Information 5 Cytoscape file. Cytoscape file containing the full networks and modules with gene and network information for the TP1, TP3, and TP5 gene co-expression results.

10.7717/peerj.12335/supp-6 Supplemental Information 6 Illumina RNA-seq data generated from *Montipora capitata*. Illumina RNA-seq data generated from *Montipora capitata* as part of this study (NCBI BioProject ID: PRJNA694677).

10.7717/peerj.12335/supp-7 Supplemental Information 7 Network output. Network size and gene expression direction of individual modules for TP1, TP3, and TP5.

10.7717/peerj.12335/supp-8 Supplemental Information 8 MAGI output at TP5, showing the highest scoring gene-metabolite interactions with a MAGI score > 5. The gene annotations, analyte identifications, MAGI scores, and reaction IDs are shown for both genes (GT5) and metabolites (MRT5) at TP5. Rows highlighted in blue indicate redox reactions. Entries in the bold text take part in the same biochemical reaction.

## Additional Information and Declarations

### Field Study Permissions

The following information was supplied relating to field study approvals (*i.e*., approving body and any reference numbers): Approval to collect coral nubbins from the waters of Kāne‘ohe Bay, HI was provided by the Division of Aquatic Resources, State of Hawaiʻi (SAP 2019-60).

### DNA Deposition

The following information was supplied regarding the deposition of DNA sequences: The novel RNA-seq data are available at NCBI BioProject: PRJNA694677. The metabolomic data are available as the supplementary information associated with this article: http://advances.sciencemag.org/cgi/content/full/7/1/eabd4210/DC1.

### Data Availability

The following information was supplied regarding data availability: The code used for the DAM + gene/metabolite co-expression networks are available at Github: https://github.com/dbsymbiosis/Construct_Networks.
