# Supplementary figures and images for: Multi-omic characterization of the thermal stress phenome in the stony coral Montipora capitata
Source: PeerJ. 2021 Nov 10;9:e12335. doi: 10.7717/peerj.12335 (PMC8590396; doi:10.7717/peerj.12335)

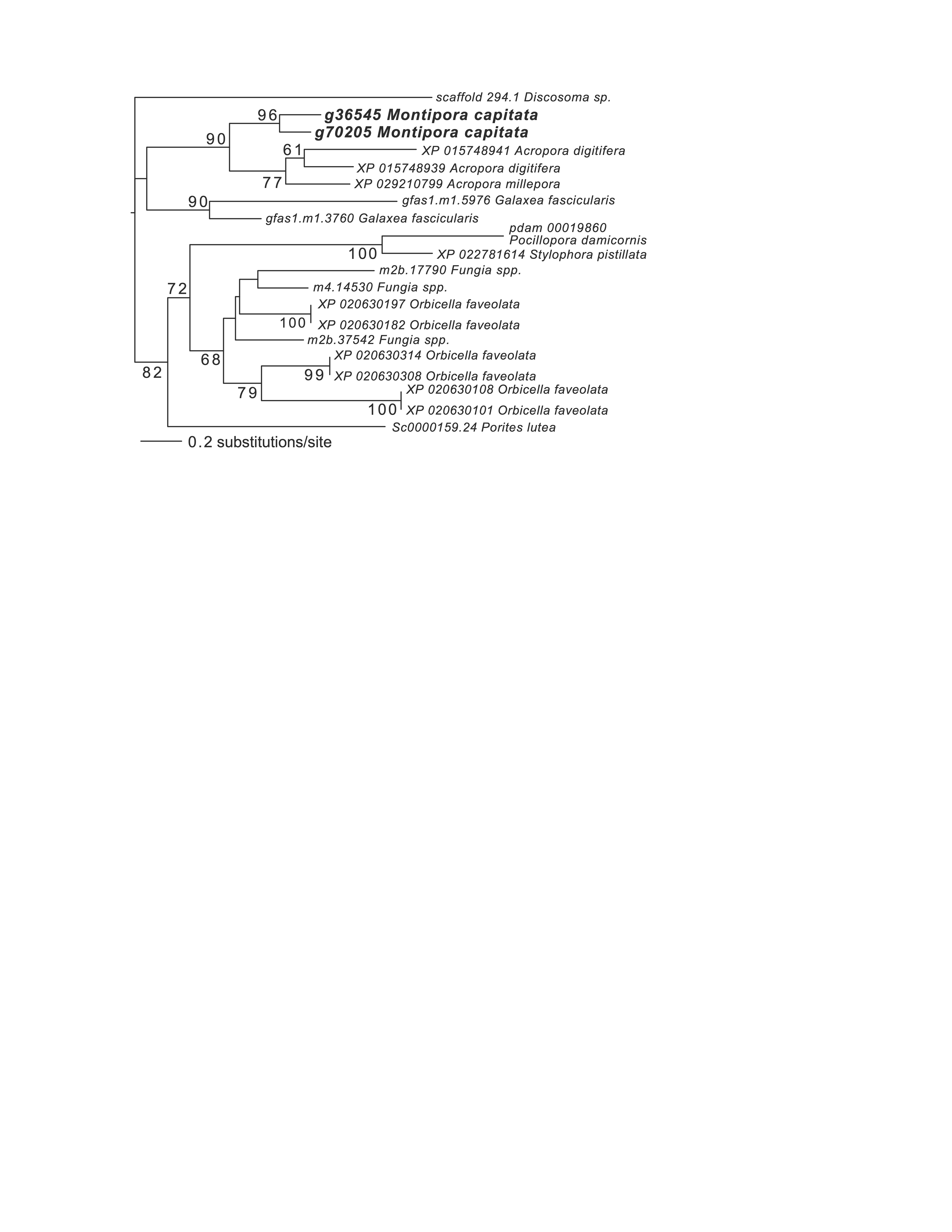

Supplement: Supplemental Information 2 — The results of the bootstrap analysis are shown on the branches when >60%. The legends show the expected substitution rate for the protein dataset. [file peerj-09-12335-s002.jpg]

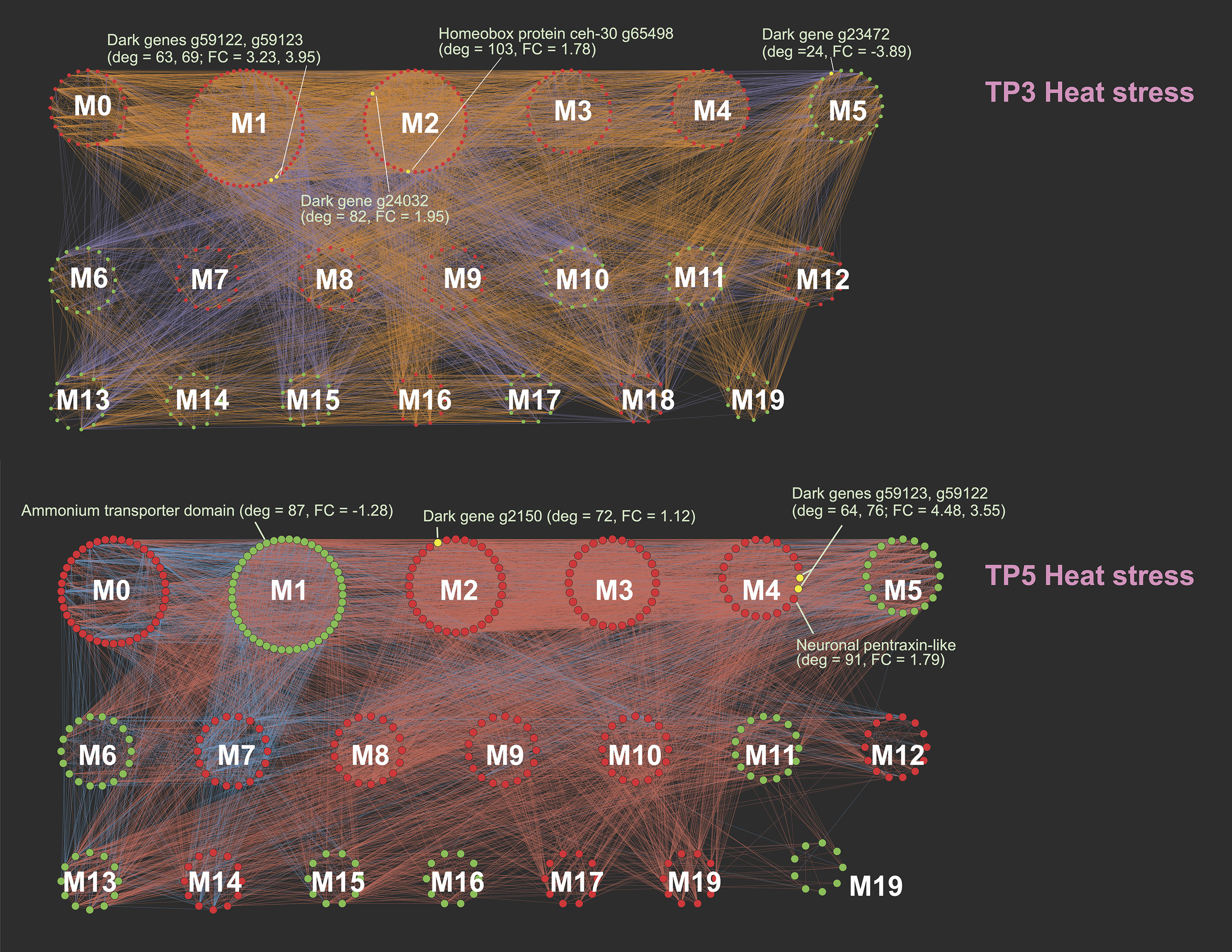

Supplement: Supplemental Information 3 — Red nodes are up-regulated, green nodes are down-regulated, and selected dark genes are the yellow nodes with gene IDs shown. The fold change (FC) and network degree value (deg) are also shown for some genes. [file peerj-09-12335-s003.jpg]

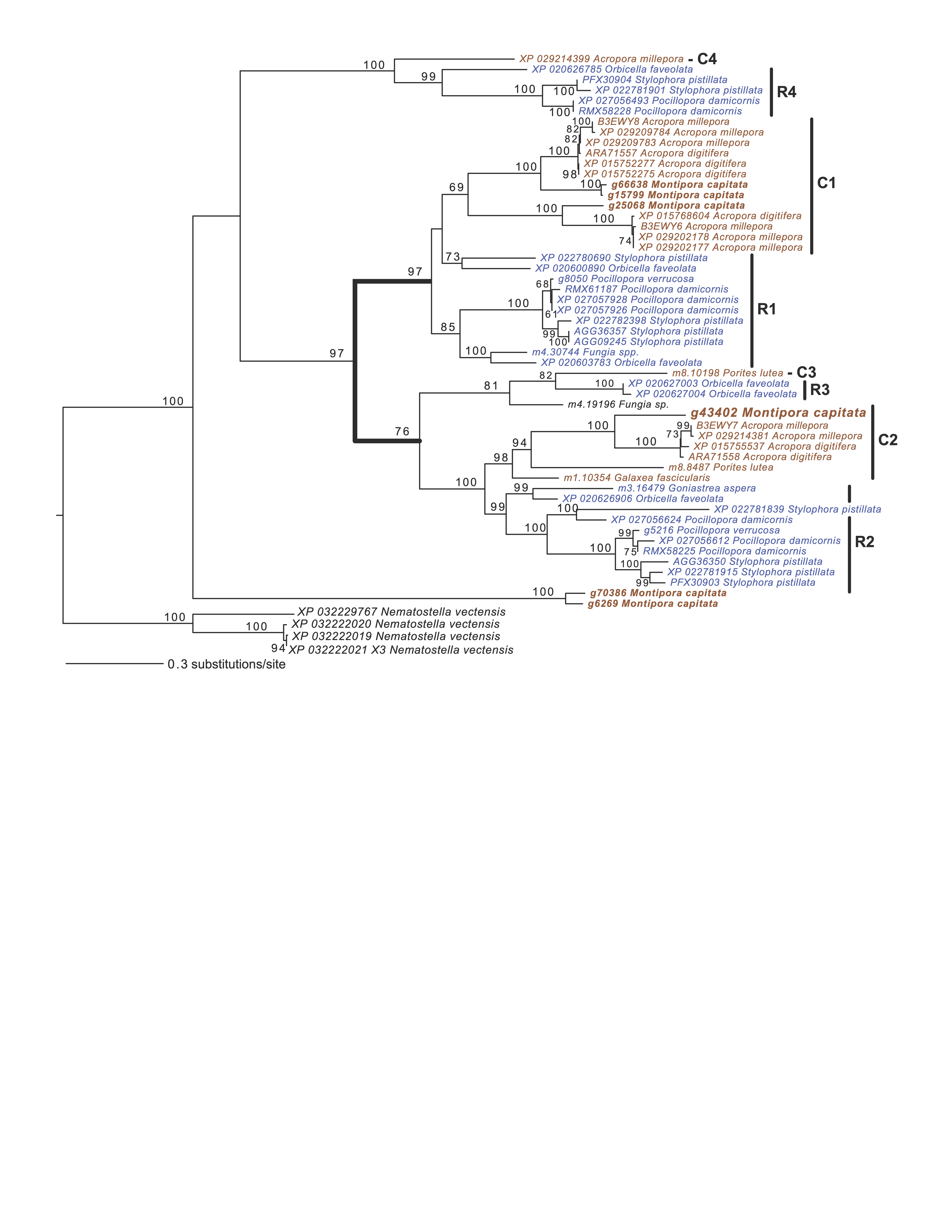

Supplement: Supplemental Information 4 — The results of the bootstrap analysis are shown on the branches when >60%. The legend shows the expected substitution rate for the protein dataset. Complex and robust coral species are shown in brown and blue text, respectively. Four putative CARP5 paralog clades are indicated. The thick branches mark a major gene duplication event in the common ancestor of complex and robust coral species. [file peerj-09-12335-s004.jpg]
