# Supplementary material for: Multi-omic characterization of the thermal stress phenome in the stony coral Montipora capitata
Source: PeerJ. 2021 Nov 10;9:e12335. doi: 10.7717/peerj.12335 (PMC8590396; doi:10.7717/peerj.12335)
Supplement: Supplemental Information 8 — The gene annotations, analyte identifications, MAGI scores, and reaction IDs are shown for both genes (GT5) and metabolites (MRT5) at TP5. Rows highlighted in blue indicate redox reactions. Entries in the bold text take part in the same biochemical reaction. [file peerj-09-12335-s008.pdf]

Table S3. MAGI output at TP5, showing the highest scoring gene-metabolite interactions with a MAGI score $\geq$ 5. The gene annotations, analyte identifications, MAGI scores, and reaction IDs are shown for both genes (GRT5) and metabolites (MRT5) at TP5. Rows highlighted in blue indicate redox reactions. Entries in the bold text take part in the same biochemical reaction.

Gene annotation	Compound name	InChI Key	MAGI score	GT5	MRT5	Reaction ID
Abhydrolase domain-containing protein 4	Isobutyric acid	KQNPFQTWMSNSAP-UHFFFAOYSA-N	5.129376	UP	UP	RXN-668
Alcohol dehydrogenase [NADP(+)]	5alpha-Androstane-3beta,17beta-diol	CBMYJHIOYJEBSB-YSZCXEEOSA-N	9.052214	UP	DOWN	RHEA:16300
Alcohol dehydrogenase [NADP(+)]	2-Hexenal	MBDOYVRWFFCFHM-SNAWJCMRSA-N	8.730280	UP	NA	RXN-12299
Alcohol dehydrogenase [NADP(+)]	Acrolein	HGINCPLSRVDWNT-UHFFFAOYSA-N	8.676901	UP	UP	RHEA:12170
Alcohol dehydrogenase [NADP(+)]	(1R,2R)-Cyclohexa-3,5-diene-1,2-diol	YDRSQRPHLBEPTP-PHDIDXHHSA-N	8.592966	UP	UP	1.3.1.20-RXN
Alcohol dehydrogenase [NADP(+)]	1-Butanol	LRHPLDYGYMQRHN-UHFFFAOYSA-N	8.548746	UP	NA	ENZRXN-201-RXN
**Alcohol dehydrogenase [NADP(+)]**	**L-Gulonic acid**	**RGHNJXZEOKUKBD-QTBDOELSSA-N**	**7.707037**	**UP**	**UP**	**GLUCURONATE-REDUCTASE-RXN**
	***aldehydo*-D-glucuronate**	**IAJILQKETJEXLJ-QTBDOELSSA-N**	**7.305923**	**UP**	**NA**	
Betaine--homocysteine S-methyltransferase 1	(2R)-2-Ammonio-4-(methylthio)butanoate	FFEARJCKVFRZRR-SCSAIBSYSA-N	10.873928	NA	DOWN	RHEA:22339
Cytochrome P450 10	(25R)-3alpha,7alpha,12alpha-Trihydroxy-5beta-cholestan-26-al	USFJGINJGUIFSY-IUFSEJPUSA-N	6.595443	NA	NA	CHOLESTANETETRAOL-26-DEHYDROGENASE-RXN
Cytochrome P450 10	(25R)-3alpha,7alpha-Dihydroxy-5beta-cholestan-26-oic acid	ITZYGDKGRKKBSN-RXDNHGQQSA-N	6.595443	UP	DOWN	RXN-9844
Cytochrome P450 1A1	6-Hydroxymelatonin	OMYMRCXOJJZYKE-UHFFFAOYSA-N	7.281551	UP	UP	RXN-11056
Cytochrome P450 1A1	Progesterone	RJKFOVLPORLFTN-LEKSSAKUSA-N	6.312111	UP	UP	RXN66-355
Cytochrome P450 3A24	Eucalyptol	WEEGYLXZBRQIMU-UHFFFAOYSA-N	8.436319	UP	UP	RXN-13133
Dimethylaniline monooxygenase [N-oxide-forming]	N,N-Dimethylaniline N-oxide	LKQUDAOAMBKKQW-UHFFFAOYSA-N	11.203145	UP	UP	RHEA:24470
DLH domain-containing protein	4-Carboxymethylenebut-2-en-4-olide	AYFXPGXAZMFWNH-UHFFFAOYSA-N	5.990086	UP	UP	RHEA:12373
FAD-linked oxidase	(R)-6-Hydroxynicotine	ATRCOGLZUCICIV-SECBINFHSA-N	6.846279	UP	UP	RHEA:10015
Glutamine amidotransferase type-1 domain-containing protein	cis-4-Hydroxy-D-proline	PMMYEEVYMWASQN-QWWZWVQMSA-N	6.300669	DOWN	NA	RXN-8003
Glutamine amidotransferase type-1 domain-containing protein	1-Aminocyclohexa-3,5-diene-1,2-diol	PWWIBLJDJCLRSC-UHFFFAOYSA-N	6.051894	UP	UP	RXN-15250
Glutamine amidotransferase type-1 domain-containing protein	N(5)-Phenyl-L-glutamine	VMNRUJGOLBSEPK-VIFPVBQESA-N	5.977851	UP	UP	RXN-15251
Guanylate cyclase (Fragment)	Pyrophosphoric acid	XPPKVPWEQAFLFU-UHFFFAOYSA-N	10.689955	DOWN	UP	RHEA:13665
Inositol oxygenase	D-Glucuronic Acid	AEMOLEFTQBMNLQ-AQKNRBDQSA-N	7.260806	UP	NA	RHEA:23697
L-tyrosine decarboxylase	Tyramine	DZGWFCGJZKJUFP-UHFFFAOYSA-N	5.953216	NA	UP	RHEA:14346
1-pyrroline-5-carboxylate synthase	D-Glutamic acid	WHUUTDBJXJRKMK-GSVOUGTGSA-N	11.232582	UP	UP	RHEA:24889
Delta-1-pyrroline-5-carboxylate dehydrogenase, mitochondrial	4-Hydroxyglutamate semialdehyde	XCXUZPXOFFRGGP-DMTCNVIQSA-N	11.232582	NA	NA	RXN-14472
**Phe-4-hydroxylase**	**Tyrosine**	**OUYCCCASQSFEME-QMMMGPOBSA-N**	**11.494889**	**NA**	**NA**	**RHEA:20273**
	**4a-Hydroxytetrahydrobiopterin**	**KJKIEFUPAPPGBC-UHFFFAOYSA-N**	**10.909811**	**DOWN**	**UP**	
	**Phenylalanine**	**COLNVLDHVKWLRT-QMMMGPOBSA-N**	**10.804993**	**NA**	**NA**	
S-(hydroxymethyl)glutathione dehydrogenase	Acetaldehyde	IKHGUXGNUITLKF-UHFFFAOYSA-N	10.629627	UP	DOWN	RHEA:25290
Glycerate dehydrogenase	D-Glyceric acid	RBNPOMFGQQGHHO-UWTATZPHSA-N	9.974234	DOWN	DOWN	RHEA:18658
